# Assessing vulnerability to risk of suicide and self-harm in prisoners: a Rasch analysis of the suicide concerns for offenders in the prison environment (SCOPE-2)

**DOI:** 10.1186/s12888-020-02569-1

**Published:** 2020-04-15

**Authors:** Amanda E. Perry, Mike Horton

**Affiliations:** 1 Department of Health Sciences, Alcuin C Block, University of York, Heslington, York, Y010 5DD England; 2 Academic Department of Rehabilitation Medicine, Faculty of Medicine and Health, University of Leeds, Martin Wing Level D, Great George Street, Leeds General Infirmary, Leeds, LS1 3EX England

## Abstract

**Background:**

With increasing levels of suicide and self-harm behaviour in the criminal justice system professionals would benefit from a tool that can identify individuals who may be at risk of self-harm and/or suicidal behaviour.

**Method:**

The Suicide Concerns for Offenders in the Prison Environment (SCOPE) tool was originally devised and validated in six UK prisons between 2003 and 2004. The goal of this study is to re-evaluate the SCOPE using Rasch methodology to produce a psychometrically robust instrument. Data were presented from 1051 SCOPE assessments of male and female offenders.

**Results:**

The analysis produced a revised SCOPE-2 tool reducing the tool from a 27 to a 19 items and simplifying the categorical six point scale to a four item scale.

**Conclusions:**

Further validation of the new SCOPE-2 tool is required in samples of male and female prisoners to assess different cut-off points for clinical and policy use.

## Background

Suicide is a worldwide phenomenon with over 800,000 people taking their lives each year and is projected to rise to 1.53 million by 2020 [1–3]. Vulnerable populations such as prisoners within society are known to be at greater risk of completed suicides; with male prisoners being 5 times higher and in female prisoners 20 times higher than in general population controls [4].

Self-harm is a major problem in the prison environment because individuals often repeat self-harm [5]. Such repetition has been shown to increase the probable risk of ultimate suicide. Recent evidence suggests that incidence of self-harm in UK prisons in the 12 months to March 2016 have increased by 27% on the previous year, an increased rate of 405 self-harm incidents per 1000 prisoners, compared with 320 incidents per 1000 prisoners in the previous year [6].

Monitoring of self-harm and suicidal behavior in England and Wales has been improved in recent years. Several initiatives, including the introduction of Safer Custody measures through the Assessment, Care in Custody and Teamwork: (ACCT) system [7] provide a mechanism for keeping prisoners safe [8]. Whilst this mechanism exists, the process by which someone is identified is problematic because of the potential for many false positive results [9]. Any such screening tool must therefore focus on trying to identify those most at risk whilst producing as few false positives as possible. This balance is important given the stringent financial and resource constraints within the system and the implications it may have for individuals who were subsequently found not to be at risk.

Systematic review evidence suggests the sparse nature of existing tools and highlights the range of limited psychometric tools containing thorough examination [9, 10]. The results of the most recent review identifies questions (i) how best to identify those most at risk in an already vulnerable population and (ii) how an individual can be accurately identified. One of the tools identified in the review was the SCOPE instrument [11].

The SCOPE was originally devised with male and female prisoners between 2003 and 2004 using traditional psychometric methods of exploratory and confirmatory factor analysis [11]. The tool was derived to assess vulnerability to risk of suicide and self-harm behaviour in prisoners (i.e., not self-harm behaviour per se) and was constructed to fulfil an evidence gap whereby previous use of existing tools (constructed with samples of *community* psychiatric patients) were used in prisoner populations.

Historically, this led to tools being used in prisons which were not contextually appropriate and meant that people were completing items on questionnaires which had a different meaning because of the prison environment (e.g., feelings of punishment and guilt as presented in the Beck Depression Inventory) resulted in higher threshold scores and high endorsement of items not previously seen in community populations [12].

The development of the SCOPE tool was perhaps unconventional in its approach to generating items for the new tool. Whereas other methods of tool construction are based on the aetiology of the health construct (e.g., items for measuring depression include symptom items asking about a lack of appetite) the SCOPE items were generated using a series of 28 different vignettes containing risk factors that were known to increase and decrease risk of suicide and non-fatal self-harm behaviour. This method devised by Forbes and Roger in 1999 has been used in the construction of other instruments whereby participants were asked to respond using a cognitive behavioural framework (e.g., [13]).

In devising the SCOPE tool the 28 participants were asked to imagine how each person would feel (emotion), how they would react (behaviour) and what they would think (cognitive). The vignette responses yielded over 1000 statements which were iteratively reduced to a pool of 92 remaining statements which reflected these responses, for example, *‘I do not think about harming myself’* (for more details on the method see Perry & Olason, 2009). Its approach has a number of advantages; most important was the fact that the items were generated by those within the prison environment producing contextually relevant responses unique to this population within the prison environment.

Nevertheless, there are several recognised limitations of the SCOPE as a self-report instrument. Results from the recent systematic review suggest that the 27 self-report Likert scale items would be time-consuming to complete in a busy reception environment, and difficult for staff to implement [10]. The scale itself comprises of six responses and forces respondents to choose a non-neutral response as there is no “neither agree nor disagree” response. It has been argued that this could potentially compel participants into presenting as either more or less at risk of harm than they actually are [10].

The utilisation of modern psychometric methodology (e.g. Rasch modeling) offers an alternative approach to further assess the psychometric properties of the SCOPE in its current format, providing a formal basis to address some of the potential psychometric limitations. Modern Test Theory provides a useful advancement to traditional psychometric methods, and it has been increasingly adopted as a means to further investigate limitations in the use and interpretation of clinical outcome measures (e.g. [14]). The modeling process provides an integrated framework to explore different measurement characteristics of a scale. This integrated approach emphasizes the relationship between the scale items and an assumed underlying latent construct, where the intention is to disclose the measurement anomalies within an item set [15]. The Rasch model [16] has a number of assumptions, including that of a unidimensional structure, and the satisfaction of these assumptions provides fundamental measurement [17]. Any deviation from this measurement structure is identified through a series of fit statistics [18]. This methodology has been used to successfully evaluate other psychiatric rating scales, including the Hamilton Rating Scale for Depression, the Patient Health Questionnaire, version-9 and the Beck Depression Inventory to improve their psychometric properties (e.g., [14, 19]).

The aim of the study was to determine the validity of the SCOPE using Rasch analysis. More specifically, the analysis process aimed to assess the psychometric properties of the 27 items within the SCOPE, and to determine the validity of the original scale structure to produce a psychometrically robust instrument. To our knowledge, this is the first study to perform Rasch analysis on the SCOPE.

## Method

### The original SCOPE sample and data collection procedures

The data for this study comes from assessments of 1166 offenders in six UK prisons collected between January 2003 and August 2004 [11]. The original data against which the SCOPE was validated were collected in study one from two prison sites (one male and one female) between January and April 2003 (*n* = 286). The administration involved a voluntary purposive cross sample of participants that were in prison on the day of administration. Administration was self-report and completed during prison ‘lock down’ over a series of successive lunchtime periods. The 92 items were generated from the vignette responses and completed using pen and paper and the responses returned anonymously for data input. Respondents are asked to circle a self-report questionnaire rated on a categorical scale of 1 to 6 from ‘strongly agree’ (1), ‘mildly agree’ (2), ‘agree’ (3), ‘disagree’ (4), ‘mildly disagree’ (5) and ‘strongly disagree’ (6), taking approximately 5 min to complete. Two subsequent studies collected further samples of responses from 486 and 406 participants across a further four sites. This data was used to assess the test re-test, concurrent and discriminate validity qualities of the tool and conduct a confirmatory factor analysis [11].

### The original data analysis procedure

Responses to the 92 items were analysed and resulted in the removal of redundant or indiscriminating items using the 80–20 split devised by Kline [20]. Remaining items were subject to principal axis factoring following a scree test, a two-factor orthogonal (Varimax) terminal solution was obtained. Using a minimum exclusion criterion of .39, 27 items loaded significantly on two factors [21]. The confirmatory factor analysis used five goodness of fit test to assess the confirmatory factor analysis of the data [22]. Following Kishtons and Widamans (1994) guidelines items were randomly allocated into six parcels three for each factor [23, 24] parcelling and item methodology were used to take into consideration problems associate with the large number of items in the confirmatory factor analysis. Each parcel was factor analysed using principle axis factoring and scree plots which showed that all parcels were one dimensional and the alpha coefficients were satisfactory. Overall, the results provided an appropriate fit for the data with four out of the five fit indices indicating an acceptable two-factor model.

### The resulting SCOPE tool

The resulting tool named ‘**S**uicide **C**oncerns for **O**ffenders in the **P**rison **E**nvironment’ (SCOPE) was a 27-item, two factor scale used to identify vulnerability to risk of self-harm and suicide in male and female offenders in the prison environment. The two- factor scale included a 15-item factor referred to as ‘Optimism’ and a 12-item factor referred to as ‘Protective self-worth’. The Optimism factor contained items evidencing low suicidal tendencies, including self-esteem, optimism and resilience. Items within the Protective self-worth factor were predominantly related to use of support networks, problem solving ability and protective factors, such as contact with family members.

The scale demonstrated moderate test re-test reliability (*n* = 115) after a 10–12 week interval (Pearson’s *r* = 0.441) and adequate internal reliability for the total scale (alpha = 0.83) and each subscale (Optimism = 0.86; Protective self-worth = 0.71) [11]. The relatively small sample of those participants completing the tool twice reflected the relatively long test re-test period and the measurement of a behaviour which will change according to an individual personal circumstances and the findings have arguably limited generalisability within these constraints [25].

The original SCOPE has good concurrent validity with other established tools which have been shown to be robust predictors of suicidal behaviour (e.g., the Beck Hopelessness Scale: BHS [26];). The results showed that people scoring higher (and therefore at greater risk) on the Optimism and Protective self-worth subscales were significantly positively correlated with feelings about the future (*r* = 0.33; *p* < 0.01, *r* = 0.42; *p* < 0.01), loss of motivation (*r* = 0.33; *p* < 0.01, *r* = 0.33; *p* < 0.01), and future expectations (*r* = 0.41; *p* < 0.01, *r* = 0.34; *p* < 0.01) respectively on the BHS. The predictive measurement of suicide and self-harm behaviour in a follow up study presented a range of sensitivity and specificity values (54.6 to 80%, and 62.2 to 69.4% respectively [27].

### Assessment using Rasch methodology

The Rasch assessment approach is appropriate where the intention of a scale or subscale is to sum all the items to form an overall score, as is the case with the two separate factors of the SCOPE. Rasch assessment methodology provides a unified framework that allows for the investigation of several aspects of internal construct validity of the item set. This range of assessments have been previously described elsewhere [18, 28], but included are assessments of: scale uni-dimensionality - whether all items are working together to measure the same construct; response dependence - whether a person’s response to an item has a direct impact on their response to any other item, after controlling for the underlying trait; response category functioning - whether the response categories of individual items represent the hierarchical structure that is assumed, with logical, ordered category thresholds; scale targeting – the relative distribution of item locations and person locations on the same underlying continuum; item bias (differential item functioning – DIF) - whether an item operates invariantly across different specific groups. e.g., males and females; and, person separation reliability index (PSI) - to examine the internal consistency reliability of the item set, including the ability of the measure to discriminate amongst persons with different levels of the underlying trait.

To investigate whether the pattern of item responses observed in the data matched the expectations of the Rasch measurement model, the two separate subscales of the SCOPE were assessed using the Rash Unidimensional Measurement Models 2030 software (RUMM 2030) [29] under a partial-credit parameterisation [30]. As data is being compared to the Rasch model, the tests-of-fit should be non-significant for the model assumptions to be satisfied. Individual items should demonstrate chi-square and Analysis of Variance (ANOVA) fit statistics > 0.05 (Bonferroni adjusted), and the same ranges are applicable for any DIF tests, which are also assessed by ANOVA. Fit residuals (z-standardised) are expected to fall within the range − 2.5 to + 2.5. A residual correlation (Q3) value of 0.2 above the average correlation was used to indicate response dependency [31]. A series of t-tests were used to assess unidimensionality, with an indication of multidimensionality (or non-unidimensionality) being apparent where the lower bound 95% CI percentage of significantly different t-tests is greater than 5% [18, 32].

### Preparation of the dataset

A total of 1166 participants obtained from the original dataset were eligible for inclusion in the study. One hundred fifteen participants were exclude due to measurement at two time points leaving a sample of *n* = 1051. This sample comprised of 59% male, with a mean age of 24.1 years (S.D. 9.48), see Table 1. The sample was randomly split into an experimental (s0) and validation sample (s1) to enable cross validation of the dataset.

**Table 1:** Description of offender sample

Age range, (Mean and SD)	Female sample (*N* = 427)	Male sample (*N* = 624)
	15–65 (29.11, 8.29)	14–66 (20.55, 8.64)
**Ethnicity N (%)**		
White British	379 (88.8)	495 (79.3)
African American	28 (6.6)	51 (8.2)
Asian	1 (0.2)	23 (3.7)
Mixed Race	7 (1.6)	9 (1.4)
Other	5 (1.2)	13 (2.1)
Missing	7 (1.6)	33 (5.3)
**Committing offence**^1^**or on remand N (%)**		
Violence against the person	27 (6.3)	56 (9)
Sexual offences	0 (0)	56 (9)
Burglary and Robbery	49 (11.5)	195 (31.3)
Theft	58 (13.6)	72 (11.5)
Fraud and forgery	7 (1.6)	14 (2.2)
Criminal damage	8 (1.9)	8 (1.3)
Drug	64 (15.0)	24 (3.8)
Motoring	13 (3.0)	59 (9.5)
Other	31 (7.3)	58 (9.3)
On remand	151 (35.4)	34. (5.4)
Missing	19 (4.4)	48 (7.7)
**At risk of self-harm N (%)**		
Current self-harm ideation	118 (27.6)	90 (14.4)
Current suicidal ideation	123 (28.8)	128 (20.5)
Historical self-harm or suicidal behaviour	61 (14.3)	43 (6.9)
No history of the above	235 (55.0)	432 (69.2)

^1^The category of offences were taken from The Offenders Index Code Book. Accessed through the UK data service

## Results

Initially, the complete 27-item set was assessed for its unidimensionality when all items are considered as a single factor. It was anticipated that the item set would fracture into the original two-factor structure within the residual principle-component loading structure.

The non-unidimensionality of the two original factors was confirmed (series of t-tests = 21.9%; CI: 20.6–23.2%), and although the anticipated loading structure was almost completely recovered, this process highlighted differential factor involvement for two of the items. Originally, item 16 ‘I enjoy everything’ was classified within the Optimism factor, but this item was shown to load alongside the Protective self-worth items. Likewise, item 19 ‘If I were depressed I would talk to someone’ was originally classified within the Protective self-worth factor, but this item was shown to load alongside the Optimism items. In isolation, these findings may have been due to random variation in the dataset, but these findings were replicated across both the experimental sample (s0) and the validation sample (s1), thus cross-validating the differential loadings.

### Factor inclusion

The Optimism factor originally comprised of 15 items, we added our cross loading to this factor item 19 was also included meaning that the Optimism item set initially comprising of 16 items. The Protective self-worth factor originally comprised of 12 items, but item 16 was also included, meaning that the Protective self-worth item set initially comprising of 13 items. The summary fit statistics of the initial analyses of each factor within each sample (s0 and s1) are presented in Table 2, under the headings Optimism – Initial, and Protective self-worth – Initial.

**Table 2:** Rasch results of the analysis on SCOPE

				Item Fit Residual		Person Fit Residual		Chi Square Interaction			PSI		Unidimensionality T-Tests (CI)	
	Analysis	Number of items	valid n	Mean	SD	Mean	SD	Value	df	p	with extrms	NO extrms	%	lower bound 95% CI
Optimism Sample 0	Initial	15 + 1	521	1.25	2.87	−0.21	1.38	393.3	144	> 0.001	0.85	0.84	5.8%	3.9%
	Final	10	518	0.71	0.74	−0.37	1.46	96.0	80	0.11	0.75	0.72	5.8%	3.9%
Optimism Sample 1	Initial	15 + 1	517	1.33	2.58	−0.21	1.47	411.5	144	> 0.001	0.86	0.84	7.2%	5.3%
	Final	10	516	0.54	1.01	−0.37	1.44	74.7	80	0.65	0.77	0.73	4.8%	3.0%
Protective Self-Worth Sample 0	Initial	12 + 1	518	1.18	2.21	−0.15	1.36	228.5	117	> 0.001	0.75	0.71	4.8%	3.0%
	Final	9	509	0.54	1.13	−0.28	1.25	90.3	81	0.22	0.65	0.60	2.4%	0.5%
Protective Self-Worth Sample 1	Initial	12 + 1	522	1.10	2.08	−0.15	1.37	246.9	117	> 0.001	0.73	0.69	5.2%	3.3%
	Final	9	511	0.68	1.55	−0.30	1.33	111.9	81	0.01	0.64	0.56	1.8%	–
	Ideal Values			0	1	0	1			> 0.05	> 0.85	> 0.85	< 5%	< 5%
											> 0.7	> 0.7		

### Rescoring – both factors

At this first stage, it was observed that disordered response category thresholds were present within both separate factors. This disorder was present across 15/16 items in the Optimism factor, and across all items in the Protective self-worth factor. This finding was identically replicated across both validation samples. The response category structure evidence suggests that respondents are not distinguishing between the response categories of ‘mildly agree’ and ‘agree’; or ‘mildly disagree’ and ‘disagree’. This finding reflects the criticisms of others who expressed concerns about the structure of the Likert scale (Gould et al. 2017). In order to address this, a generic recode was implemented, where all items were recoded to effectively treat the ‘mildly agree’ and ‘agree’ categories as equivalent, and the ‘mildly disagree’ and ‘disagree’ categories as equivalent, meaning that each item now had four implied response categories, rather than the original six that were presented (see Appendix 1).

### Assessment process

Following the generic rescore, within each separate factor all items were assessed individually and collectively for any source of misfit, including departures from the probabilistic structure, response dependency, and DIF by age, gender and remand status. All potential indications of misfit were cross-checked across the separate validation samples to ensure consistency, and options to address the anomalies were considered. In order to refine the item set to create a psychometrically robust instrument, items displaying misfit anomalies were iteratively removed, with the remaining item set being re-assessed following each removal.

### Optimism factor refinement

For the Optimism factor, this process resulted in the removal of six items:

1. Items 6 (I do not think about harming myself),
2. Item 7 (I do not feel suicidal when I receive bad news)
3. Item 25 (If I were depressed I would not think about harming myself) were removed as they formed a dependent cluster alongside Item 21 Item 21 (I do not think about how I can end my life), which was retained in the final item set.

Each one of these items independently operates well alongside the other nine items in the final set, but dependency remains when any combination of more than one item is included.

4. Item 13 (I do not feel fed up) was removed due to the consistent display of an over discrimination misfit anomaly.
5. Item 16 (I enjoy everything) was removed due to the consistent display of a large under discrimination misfit anomaly.
6. Item 17 (I do not feel helpless) was removed due to the consistent display of an over-discrimination misfit anomaly, plus a suggested dependency with item 14 (I think that everyone likes me).

At this stage, all items fit individually and at the scale level leaving ten items remaining in the final Optimism item set. There was no evidence of dependency or multidimensionality. The revised rescoring structure (mildly and agree together/ mildly disagree and disagree together) appeared to work well across all items except for item 21 (I do not think about how I can end my life) and item 24 (I feel like there is hope in my life). Item 21 appears to lend itself to a dichotomous structure, so this amendment was implemented in the ‘Final’ analysis. Item 24 remained slightly disordered, but no adjustment was made at this point. The summary fit statistics of the final item set within each sample (s0 and s1) are presented in Table 2, under the heading ‘Optimism – Final’.

### Protective self-worth refinement

For the Protective self-worth factor, this process resulted in the removal of four items:

1. Item 4 (I will speak to an officer when I have a problem) was removed due to the consistent display of dependency with item 3 (If I were feeling suicidal I would speak to someone). Item 3 was retained as preferential due to the additional information regarding suicidal thoughts, which may be useful for prison management.
2. Item 9 (If I had been arrested I would try and get in contact with my family) was removed due to the consistent display of dependency with item 20 (My family support me). Both of these items reflect family support, but item 20 was retained as preferential as it is semantically clearer, and it is both conceptually and psychometrically stronger
3. Item 16 (I enjoy everything) was removed due to the consistent display of dependency with item 15 (The day before I am due in court I do not think about the future). Item 15 was retained as preferential as it was thought that item 16 is too general for the underlying concept that is being targeted.
4. Item 19 was removed due to the consistent display of a large under discrimination misfit anomaly.

At this stage, all items fit individually and at the scale level leaving nine items remaining in the final Protective self-worth item set. There was no evidence of dependency or multidimensionality. The revised rescoring structure appeared to work well across all items except for item 20 (My family support me) and item 26 (If I had a supportive family I would not kill myself), where a dichotomous structure was suggested. Again, this amendment was implemented in the ‘Final’ analysis.

The summary fit statistics of the final item sets within each sample (s0 and s1) are presented in Table 2, under the heading Protective ‘self-worth – Final’.

### DIF

Alongside the other tests of fit, each item was assessed for DIF by gender (male Vs female), age group (adult Vs young offender), and remand status (on remand Vs sentenced) across each of the factors. In the final item sets some inconsistent item DIF was indicated for each of the person factors across the samples, which may potentially signify a degree of item bias. However, more noteworthy was the DIF that was observed consistently across both samples.

In the Optimism item set, this consistent DIF was observed age group for item 1 ‘I do not feel lonely in my room on my own’, with adults reporting that they would be more likely to agree with this statement than young offenders across all underlying levels of the latent trait. There was no consistent DIF indicated by gender or remand status.

In the Protective self-worth item set, this consistent DIF was observed by gender for item 22 ‘If I had a fight with a prisoner I would ask to see the governor’, with females reporting that they would be more likely to agree with this statement than males across all underlying levels of the latent trait. Additionally, consistent DIF was observed by age group for item 10 ‘When arrested I would say I was sorry’, with adults reporting that they would be more likely to agree with this statement than young offenders across all underlying levels of the latent trait. Again, there was no consistent DIF indicated by remand status.

### Final stage

When each of these final ‘pure’ item sets had been identified, each removed item was individually re-introduced alongside the final item set, in order to assess whether the initial misfit anomaly (and reason for the item’s removal) remained. This remained the case for all removed items, meaning that the final Optimism item set remained as ten items, and the Protective self-worth item set remained as nine items (see Appendix 1).

**Fig. 1:**
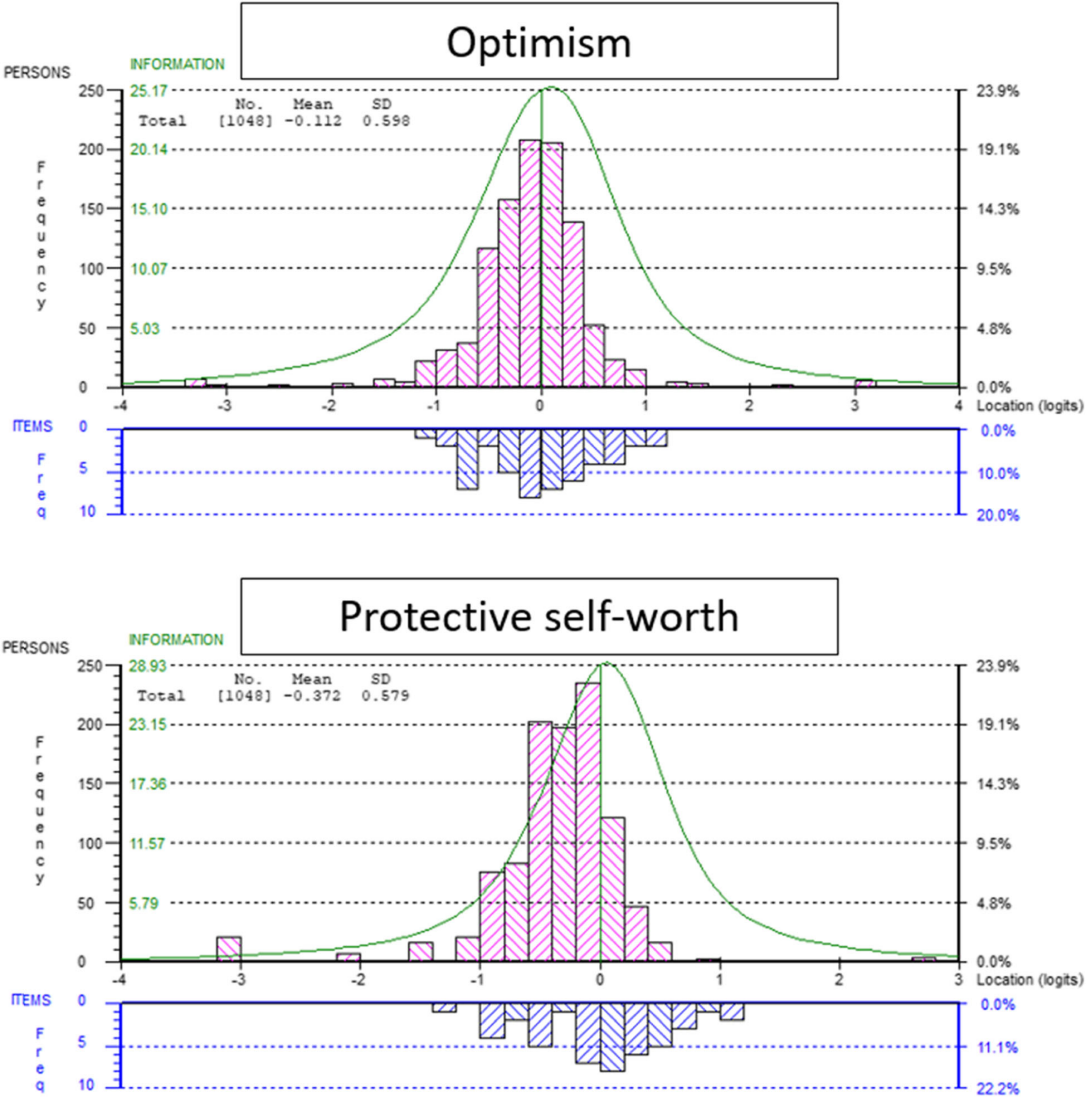
Person-item threshold distribution, displaying the relative logit location distribution of the final Optimism and Protective self-worth item sets within an offender population

Additionally, the final two factor-item sets were again investigated in the context of a single item set among the full sample. This confirmed the distinct separation of the two concepts, as non-unidimensionality was confirmed (series of t-tests = 17.8%; CI: 16.5–19.1%). A subsequent subtest analysis of the two factors revealed their latent correlation to be r = 0.217, offering support for the distinct separation of the two concepts. The original and new factor structure of the SCOPE are presented in Table 3, where the reasons for item removal are also summarised. The final item fit statistics for the two separate ‘Optimism’ and ‘Protective self-worth’ item sets are presented in Table 4, and the targeting of the scales to the sample is presented in Fig. 1

**Table 3:** SCOPE: factor structure and refinement

Original Item No.	New Item No.^1^	Statement	Original Factor^2^		Rasch Refinement
			Optimism	PSelf-Worth^3^	
7		I do not feel suicidal when I receive bad news	X		Removed due to dependency with items 6, 21 & 25
17		I do not feel helpless	X		Removed due to over discrimination misfit
25		If I were depressed I would not think about harming myself	X		Removed due to dependency with items 6, 7 & 21
18	O1	If I worry about things I sleep OK	^a^		Remains in Factor 1
13		I do not feel fed up	X		Removed due to over discrimination misfit
16		I enjoy everything	X		Removed due to under discrimination misfit
6		I do not think about harming myself	X		Removed due to dependency with items 7, 21 & 25
21	O2	I do not think about how I can end my life	^a^		Rescoring suggests dichotomy. Remains in Factor 1
5	O3	If I were on remand I would not feel stressed out	^a^		Remains in Factor 1
24	O4	I feel like there is hope in my life	^a^		Remains in Factor 1
23	O5	I can think straight when I am depressed	^a^		Remains in Factor 1
8	O6	I feel fine about coming into this establishment	^a^		Remains in Factor 1
14	O7	I think that everyone likes me	^a^		Remains in Factor 1
11	O8	If I am nervous I do not lose my appetite	^a^		Remains in Factor 1
1	O9	I do not feel lonely in my room on my own	^a^		Remains in Factor 1
19	O10	If I were depressed I would talk to someone	^a^	0	Originally in Factor 2, loads and fits with Factor 1
4		I will speak to an officer when I have a problem		X	Removed due to dependency with item 3
10	P1	When arrested I would say I was sorry		^a^	Remains in Factor 2
3	P2	If I were feeling suicidal I would speak to someone		^a^	Remains in Factor 2
15	P3	The day before I was due to appear in court I would think about the future		^a^	Remains in Factor 2
27	P4	I always turn up in court		^a^	Remains in Factor 2
9		If I had been arrested I would try and get in contact with my family		X	Removed due to dependency with item 20
2	P5	If I had a job I would not commit crime		^a^	Remains in Factor 2
12	P6	If I stole money for drugs I would feel like I had let myself down		^a^	Remains in Factor 2
22	P7	If I had a fight with a prisoner I would ask to see the governor		^a^	Remains in Factor 2
20	P8	My family support me		^a^	Rescoring suggests dichotomy. Remains in Factor 2
26	P9	If I had a supportive family I would not kill myself		^a^	Rescoring suggests dichotomy. Remains in Factor 2

^1^ New item numbering presented by factor structure o = optimism on factor one and *p* = protective self-worth on factor two

^2^ X = item removed from dataset, ^a^ = Item remains in original factor, 0 = Item switched from one factor to another

^3^ PSelf Worth = Protective Self-Worth

**Table 4:** Individual item fit of final Optimism and Protective Self-Worth item sets across two validation samples

			Location (logits)		Fit Residual (z-value)		Chi-square *P*-value		ANOVA *P*-value	
	Item	Statement	Sample 0	Sample 1	Sample 0	Sample 1	Sample 0	Sample 1	Sample 0	Sample 1
Optimism	1	I do not feel lonely in my room on my own	0.135	0.308	1.773	1.831	0.086	0.143	0.094	0.163
	5	If I were on remand I would not feel stressed out	−0.444	− 0.54	− 0.087	− 0.869	0.417	0.274	0.326	0.137
	8	I feel fine about coming into this establishment	−0.279	− 0.147	0.208	− 1.224	0.232	0.450	0.177	0.234
	11	If I am nervous I do not lose my appetite	−0.003	0.041	0.288	0.788	0.503	0.916	0.374	0.900
	14	I think that everyone likes me	−0.045	0.026	1.177	0.915	0.936	0.813	0.930	0.788
	18	If I worry about things I sleep OK	−0.012	0.01	−0.459	0.526	0.319	0.437	0.203	0.409
	19	If I were depressed I would talk to someone	−0.477	−0.589	0.618	1.814	0.419	0.229	0.379	0.279
	21	I do not think about how I can end my life	1.079	1.029	0.723	0.457	<.005^a^	0.718	<.005^a^	0.688
	23	I can think straight when I am depressed	−0.607	−0.714	1.262	0.01	0.800	0.166	0.815	0.114
	24	I feel like there is hope in my life	0.653	0.576	1.617	1.148	0.605	0.992	0.736	0.999
Protective Self-Worth	2	If I had a job I would not commit crime	0.021	−0.036	0.678	2.677^a^	0.776	0.145	0.788	0.211
	3	If I were feeling suicidal I would speak to someone	−0.06	−0.089	1.143	1.83	0.275	0.966	0.278	0.973
	10	When arrested I would say I was sorry	−0.446	−0.515	−0.582	−1.907	0.148	<.005^a^	0.093	<.005^a^
	12	If I stole money for drugs I would feel like I had let myself down	0.147	0.037	−1.652	0.14	0.034	0.544	0.009	0.611
	15	The day before I was due to appear in court I would think about the future	0.109	−0.004	0.102	0.836	0.527	0.706	0.507	0.801
	20	My family support me	0.522	0.706	1.566	1.57	0.325	0.148	0.335	0.121
	22	If I had a fight with a prisoner I would ask to see the governor	−0.901	−0.885	1.238	1.316	0.892	0.313	0.903	0.291
	26	If I had a supportive family I would not kill myself	0.64	0.801	1.975	1.291	0.256	0.590	0.256	0.592
	27	I always turn up in court	−0.033	−0.014	0.429	−1.608	0.514	0.028	0.615	0.012

^a^ item misfit indicated – in all cases this is inconsistent between samples

## Discussion

The purpose of this paper was to apply use of Rasch methodology to validate the psychometric properties of the SCOPE tool. To our knowledge this is the first study to investigate the validity of the SCOPE using this technique. The SCOPE is recognised as a reliable and valid tool using traditional psychometric techniques [11]. However, the Rasch methodology analysis has provided an opportunity for refinement of the tool revealing a number of limitations with the structure of the SCOPE and in particular the categorical scoring system as recognised by Gould and colleagues [10].

The findings of the study have shown that modifying the structure of the SCOPE has improved its format and demonstrated a good fit to the Rasch model, reducing the tool from a 27 item to a 19 item tool and reducing the categorical responses from a six to a four point scale. These adaptations will likely only improve the utility of the tool in a busy prison environment. The collapsed response structure appears to work well across almost all items of both factors. However, this would need re-testing empirically with the newly suggested response format in place, as the amendments that have been made are all post-hoc, rather than addressing the actual responses that are presented to respondents (see Table 2).

Although some DIF was indicated in each of the item sets, it was decided that nothing should be done about it at this stage due to the context in which it has been observed. It is noted that if the source of DIF involves an aspect of the item that is relevant to the content of the variable (as is the case here), then it is dubious to resolve the item in a way that would reduce the difference between the groups that are being assessed [33, 34]. Additionally, if the DIF were to be addressed through item removal, then this would result in a loss of information which would certainly be detrimental to the scale. Alongside this contextual aspect, there may be some confounding interaction between the three DIF factors, but this was not explored.

Recommendations for future use of the SCOPE-2 include a possible exploration of the SCOPE-2 as a dichotomous screening tool (thus reducing the four categorical responses to a two categorical response scale ‘agree’ or ‘disagree’ statement. Use of SCOPE-2 as a monitoring tool alongside the UK current ACCT system could help follow change in risk for an individual who might have previously which would provide opportunity for further validation purposes.

## Conclusions

Rasch analysis enabled the psychometric properties of the SCOPE to be examined in more detail that traditional psychometric approaches. The study highlighted important limitations of the SCOPE, primarily the response categories were not being used as intended and there was an overlap between some items creating a series of dependencies in this sample of prisoner responses. Further implications for research concerning the frequency of items is needed using to determine if similar limitations exist in other samples and to assess the influence of altering response category labels to uncover the potential of a total SCOPE frequency score. In particular testing in both male and female samples are required as gender difference might produce variation in cut-off scores for future benchmarking, clinical and policy-based judgements.

## Supplementary information

**Additional file 1.** Instrument to Assess Suicide Concerns for Offenders in the Prison Environment (SCOPE-2).

## Data Availability

Requests to obtain copies of the datasets used and/or analyzed during the current study will be considered by the corresponding author on reasonable request.

## Abbreviations

SCOPE: Suicide concerns for offenders in the prison environment
ACCT: Assessment care in custody and teamwork
BHS: Beck hopelessness scale
DIF: Differential item functioning
PSI: Person separation reliability index
RUMM2030: Rash unidimensional measurement models 2030
ANOVA: Analysis of variance
