# Supplementary material for: Assessing vulnerability to risk of suicide and self-harm in prisoners: a Rasch analysis of the suicide concerns for offenders in the prison environment (SCOPE-2)
Source: BMC Psychiatry. 2020 Apr 15;20:164. doi: 10.1186/s12888-020-02569-1 (PMC7161173; doi:10.1186/s12888-020-02569-1)
Supplement: Supplementary file 1 — Additional file 1. Instrument to Assess Suicide Concerns for Offenders in the Prison Environment (SCOPE-2). [file 12888_2020_2569_MOESM1_ESM.docx]

| Name:………………………………….. Date:………………………… | Gender: …………Male…………Female | | | | Score/sub-scale total |
| --- | --- | --- | --- | --- | --- |
| Are you on remand?..........Yes……….No | Age:……………………………………. | | | |  |
| PLEASE READ THE STATEMENT AND CIRCLE THE RESPONSE | Strongly Agree | Agree | Disagree | Strongly Disagree |  |
| O1. If I worry about things I sleep OK | 1 | 2 | 3 | 4 |  |
|  |  |  |  |  |  |
| O2. I do not think about how I can end my life | 1 | 2 | 3 | 4 |  |
|  |  |  |  |  |  |
| O3. If I were on remand I would not feel stressed out | 1 | 2 | 3 | 4 |  |
|  |  |  |  |  |  |
| O4. I feel like there is hope in my life | 1 | 2 | 3 | 4 |  |
|  |  |  |  |  |  |
| O5. I can think straight when I am depressed | 1 | 2 | 3 | 4 |  |
|  |  |  |  |  |  |
| O6. I feel fine about coming into this establishment | 1 | 2 | 3 | 4 |  |
|  |  |  |  |  |  |
| O7. I think that everyone likes me | 1 | 2 | 3 | 4 |  |
|  |  |  |  |  |  |
| O8. If I am nervous I do not lose my appetite | 1 | 2 | 3 | 4 |  |
|  |  |  |  |  |  |
| O9. I do not feel lonely in my room on my own | 1 | 2 | 3 | 4 |  |
|  |  |  |  |  |  |
| O10. If I were depressed I would talk to someone | 1 | 2 | 3 | 4 |  |
|  |  |  |  |  |  |
| ***Subscale total score for Optimism factor***  = | | | | | |
| P1. When arrested I would say I was sorry | 1 | 2 | 3 | 4 |  |
|  |  |  |  |  |  |
| P2. If I were feeling suicidal I would speak to someone | 1 | 2 | 3 | 4 |  |
|  |  |  |  |  |  |
| P3. The day before I was due to appear in court I would think about the future | 1 | 2 | 3 | 4 |  |
|  |  |  |  |  |  |
| P4. I always turn up in court | 1 | 2 | 3 | 4 |  |
|  |  |  |  |  |  |
| P5. If I had a job I would not commit crime | 1 | 2 | 3 | 4 |  |
|  |  |  |  |  |  |
| P6 If I stole money for drugs I would feel like I had let myself down | 1 | 2 | 3 | 4 |  |
|  |  |  |  |  |  |
| P7 If I had a fight with a prisoner I would ask to see the governor | 1 | 2 | 3 | 4 |  |
|  |  |  |  |  |  |
| P8 My family support me | 1 | 2 | 3 | 4 |  |
|  |  |  |  |  |  |
| P9 If I had a supportive family I would not kill myself | 1 | 2 | 3 | 4 |  |
|  |  |  |  |  |  |
| ***Subscale total score for Protective Self-Worth factor***  = | | | | | |
